# Anger and Aggression in UK Treatment-Seeking Veterans with PTSD

**DOI:** 10.3390/healthcare6030086

**Published:** 2018-07-21

**Authors:** David Turgoose, Dominic Murphy

**Affiliations:** 1 Combat Stress, Surrey KT22 0BX, UK; 2 King’s Centre for Military Health Research, King’s College London, London WC2R 2LS, UK; dominic.murphy@combatstress.org.uk

**Keywords:** military, veterans, anger, aggression, PTSD, mental health

## Abstract

Prevalence rates of anger and aggression are often higher in military personnel. Therefore, it is important to understand more about why this is, and the factors with which it is associated. Despite this, there is little evidence relating to anger and aggression in UK veterans who are seeking treatment for mental health difficulties such as post-traumatic stress disorder (PTSD). This study investigated the prevalence rates of anger and aggression in this population, as well as the associations between anger and aggression, and various sociodemographic, functioning and mental health variables. A cross-sectional design was used, with participants completing a battery of self-report questionnaires. Prevalence rates for significant anger and aggression were 74% and 28% respectively. Both women and those over 55 were less likely to report difficulties. Those with high levels of PTSD and other mental health difficulties were more likely to report anger and aggression. Other factors related to anger and aggression included unemployment due to ill health, and a perceived lack of family support. Findings showed that veterans who are seeking support for mental health are likely to be experiencing significant difficulties with anger and aggression, especially if they have comorbid mental health difficulties. The associations between anger, aggression, and other variables, has implications for the assessment and treatment of military veterans.

## 1. Introduction

Research has suggested that military personnel are likely to experience difficulties with anger and aggressive behaviours [1,2]. Prevalence rates in military populations have been estimated at 29% for all types of physical assault, 12% for violent behaviour, and 10% for physical assault [1,3]. Anger in military populations is strongly associated with a range of other variables, including mental health issues such as post-traumatic stress disorder (PTSD) [4]. Specifically, anger and aggression in US veterans have been associated with PTSD hyperarousal [5], PTSD re-experiencing [6], and depression [7]. The relationship between anger and mental health is complex, and there is considerable overlap, with some research showing that anger in military personnel is substantially accounted for by mental ill health [2].

Anger and aggression have also been related to an individual’s history, such as childhood adversity, childhood antisocial behaviour [2], and issues relating to their military service, such as having a combat role and experiencing multiple traumas whilst on deployment [1,2]. The extent of anger and aggression problems in military personnel is concerning given the challenges that this population faces in readjusting following deployment [8], with ex-service personnel often over-represented in prisons for violent offences and more likely to report committing violent crimes after combat exposure [9,10].

There have been several large-scale studies of anger and aggression using general military samples, often including those who have recently served in Iraq and Afghanistan. However, comparatively little research has investigated the specific mental health needs of military veterans who are seeking help for such difficulties. A recent study showed that 46% of UK veterans waited for more than five years to seek help for their mental health difficulties, and that this was related to greater mental health difficulties [11], strengthening the notion that there can be a long delay between deployment and veterans seeking support for their mental health [12]. In this sample, the second most commonly reported mental health issue was anger (76%), which has been strongly correlated with PTSD in US and UK veterans [5,6,7,11]. Given the apparently high prevalence rate of anger in treatment-seeking veterans, and the potential implications for veterans’ well-being, it is important to further our understanding of these issues to help shape mental health services and improve treatments for veterans. By investigating factors associated with anger and aggression, clinicians could be assisted in identifying risk factors in veterans seeking support for mental health difficulties.

The aims of the present study were firstly to investigate prevalence rates of anger and aggression in a sample of treatment-seeking UK veterans. Secondly, we explored the relationships between anger and aggression, and a range of sociodemographic, functioning and mental health variables. Given the past evidence showing rates and associations of anger and aggression in the wider military population, it is pertinent to investigate such links in treatment-seeking veterans as our understanding of this group is limited.

## 2. Materials and Methods

### 2.1. Procedure

A cross-sectional design was used, with questionnaire responses collected from a random sample of treatment-seeking UK veterans recruited from Combat Stress (CS), a national charity providing specialist mental health services to military veterans. Questionnaire data was collected pertaining to anger and aggression, as well as a number of mental health and sociodemographic variables.

The questionnaire contained instructions informing participants that participation was voluntary, that the research was being conducted independently from clinical services at CS, and that participant input would not affect their treatment in any way. Questionnaires were sent to participants in the post using a three-wave mail out strategy between April and August 2016. Individuals from whom a response was not received were followed up by telephone. A research assistant made three attempts to contact these individuals by telephone.

### 2.2. Participants

Data for the present study was taken from a wider investigation of the needs of treatment-seeking veterans [11]. A sample of participants was randomly taken from a population of veterans who had sought support from CS over a 12-month period, between 31st January 2015 and 1st February 2016. The sample was drawn from the total number of veterans who had attended an initial assessment and at least one further appointment during this period (*N* = 3335). From this group, a random 20% sample was taken (*N* = 667), 67 of whom were removed prior to data collection either due to participant death or not having sufficient contact information. The final sample size was 600. Of these, 403 (67%) were recruited into the study by returning completed questionnaires. As demonstrated in a previous paper, there were no significant differences between those who took part in the study and those who did not [11].

### 2.3. Outcomes

#### 2.3.1. Anger and Aggression

Data on anger was collected using the *Dimensions of Anger Reactions* measure (DAR-5) [13]. This five-item measure gives an overall score to assess anger, with items including: ‘I often find myself getting angry at people or situations’, and ‘When I get angry, I get really mad’. Items are scored on a Likert scale ranging from 0–4 and a total score is calculated by adding these together, with scores of 12 and above indicating significant difficulties with anger.

In order to assess aggressive behaviours, we used a measure developed by the Walter Reed Army Institute of Research [14], based on the Interpersonal Conflict Scale [15] and the State/Trait Anger Scale [16]. This measure has been used previously in military samples [2,14]. For the purposes of this study, we termed this four-item measure the *Walter Reed Four* (WR-4). The WR-4 included the following items: ‘How often did you get angry at someone and yell or shout’, ‘How often did you get angry with someone and kick or smash something, slam the door, punch the wall etc.’, ‘How often did you get into a fight with someone not in your family and hit the person’ and ‘How often did you threaten someone with physical violence’. Respondents were asked to rate each question with five options (never, once, twice, three or four times or five or more). These were scored between 0–4 and a total score calculated by adding these together. Caseness was defined if participant total scores were in the highest tertile.

#### 2.3.2. Socio-Demographic Outcomes

Participants completed questionnaires relating to sociodemographic variables, including age, sex, relationship status and employment status. Participants were also asked to state how many years had passed between leaving the Armed Forces and seeking help. This was divided into <5 years and >5 years, and the date they left service, which was used to determine if they were an early service leaver, defined as leaving with under four years of continuous service.

Data was collected on childhood adversity, whereby participants rated 16 true or false statements relating to events from childhood, e.g., ‘I regularly used to see or hear physical fighting or verbal abuse between my parents’. These items were taken from a previous epidemiological study of health and well-being in the UK military [17]. Participants responded on a binary yes/no scale to each item. Total scores were added and those in the top tertile deemed as having high levels of childhood adversity.

#### 2.3.3. Functioning

A number of factors relating to general functioning were measured, including relationship and employment status. Data was also collected about social support, with participants asked to complete items on whether they felt supported by friends and family members.

The *Work and Social Adjustment Scale* (WSAS) [18] was used as a basic measure of functional impairment, i.e., the extent to which health difficulties interfere with the ability to carry out day-to-day tasks such as work and relationships. Totalled scores on this measure are categorised as mild (1–10), moderate (11–20) or severe (21+).

#### 2.3.4. Health

A number of mental health outcomes were assessed. PTSD was measured using the *PTSD Checklist* [19] (PCL-5); a validated, 20-item measure assessing all domains of PTSD. Items are scored on a Likert scale from 0–4, with total scores of 34+ indicating caseness for PTSD. Common mental health difficulties (CMD) of anxiety and depression were assessed using the 12-item *General Health Questionnaire-12* [20] (GHQ-12). Scores on the GHQ-12 range from 0–12, with caseness defined as a score of 6+.

Data on alcohol use was collected using the *Alcohol Use Disorders Identification Test* [21] (AUDIT). This ten-item measure gives an overall score to assess alcohol-related risk. Harmful drinking levels were defined by scores of 16+.

Traumatic brain injury (TBI) was measured using the *Brain Injury Screening Index* [22]. Participants were deemed to meet criteria for TBI if they reported experiencing a serious blow to the head plus one of a series of symptoms as a result, such as alteration of mental state (e.g., dazed), gaps in memory of over one hour, or loss of consciousness.

### 2.4. Analysis

The first stage of the analysis was to calculate prevalence rates for the DAR-5 and WR-4. Following this, logistical regression models were fitted to explore associations between the DAR-5 and WR-4, and sociodemographic factors. These were adjusted for variables found to be significant (age, sex and childhood adversity) in order to control for any mediating or moderating effects. This analysis was repeated to explore associations between the DAR-5 and WR-4, and outcomes of functioning and health. All analyses were conducted using STATA 13.0 (College Station, TX, USA).

## 3. Results

### 3.1. Demographics

Of the sample of 600 veterans who were sent the questionnaire, 403 (67.2%) responded. The majority were male (95.8% vs. 4.2% female). Most participants were over 45 years old (68.2%), and just over two-thirds of participants were unemployed (68.1%). More were currently in a relationship (60.8%) than not (39.2%). Regarding their time spent in the Armed Forces, 12.6% were classified as early service leavers, and 45.7% had a period of five years or more since leaving the military and seeking help from CS. Based on established cut-off scores, 74% of participants reported significant difficulties with anger on the DAR-5. For aggressive behaviours, 28% of participants reported significant difficulties as indicated by scores on the WR-4.

### 3.2. Relationships between Anger, Aggression and Other Variables

#### 3.2.1. Sociodemographic Variables

Table 1 contains sociodemographic variables and their associations with anger and aggression. Although only a small minority of participants were female, results suggest that women were less likely to report issues with anger (DAR-5: Odds Ratio 0.34, 95% Confidence Interval 0.12–0.92). Similarly, participants who were over the age of 55 were less likely to report aggressive behaviours (WR-4: OR 0.34, 95% CI 0.16–0.72). Participants who reported a high number of childhood adversity events were more likely to report difficulties with both anger and aggression (DAR-5: OR 3.43, 95% CI 1.68–7.00; WR-4: OR 2.22, 95% CI 1.34–3.69), although this association may be explained by other variables, such as PTSD (see Table 1).

#### 3.2.2. Functioning

Table 2 presents functioning variables and their associations with anger and aggression. Social support was a significant factor, with those reporting that they did not feel supported by their family more likely to report problems with aggressive behaviours (WR-4: OR 3.10, 95% CI 1.59–6.01). This was not replicated in those not feeling supported by friends. Employment status was also a significant factor, with unemployment due to ill health associated with higher rates of both anger and aggression (DAR-5: OR 2.80, 95% CI 1.58–4.96; WR-4: OR 2.62, 95% CI 1.51–4.55). Unemployment not due to ill health was not a significant factor. Also, those participants who reported severe levels of functional impairment were more likely to be experiencing high levels of both anger and aggression (DAR-5: OR 2.89, 95% CI 1.77–4.74; WR-4: OR 1.01, 95% CI 1.00–2.79), although this association may be explained by other variables, such as PTSD (see Table 2).

#### 3.2.3. Health

Table 3 presents associations between anger, aggression and different health variables. The most strongly associated variable was PTSD, with those meeting case criteria for PTSD being more likely to report difficulties with both anger and aggressive behaviours (DAR-5: OR 10.70, 95% CI 5.79–19.60; WR-4: OR 8.71, 95% CI 2.99–25.40). The same associations were true of common mental health difficulties such as depression and anxiety (DAR-5: OR 4.14, 95% CI 2.47–6.94; WR-4: OR 6.00, CI 2.97–12.10), and difficulties with alcohol misuse (DAR-5: OR 2.08, 95% CI 1.08–4.01; WR-4: OR 2.05, 95% CI 1.21–3.47).

Further analysis was conducted to explore these associations, whilst adjusting for all other significant variables, to see which variables were still significant after controlling for all others. Findings are presented in Table 4. Childhood adversity, severe functional impairment and being single were no longer associated with anger or aggression following these adjustments.

## 4. Discussion

This study observed that nearly three quarters of treatment-seeking veterans in this sample reported significant difficulties with anger, and more than a quarter reported problems with aggressive behaviour. Furthermore, anger and aggression were strongly associated with PTSD, and also associated with common mental health difficulties, and alcohol misuse. Links between anger and PTSD and other mental health disorders have been found previously [4], but this is the first study to show this relationship in veterans who are seeking help for their mental health.

One possible explanation may be that some PTSD, depression or anxiety symptoms overlap with anger and aggression. For example, irritability is a common feature of PTSD, which, given the association between anger and PTSD, raises the question of whether anger in treatment-seeking veterans is a separate construct, or can be explained by its relation to other mental health difficulties. Indeed, past research has suggested that the strong association between anger and mental health difficulties is due to the overlap of these conditions within the individual [2].

Previous research has found that after deployment, military personnel who experienced more traumatic events had higher levels of anger [1], which might partly explain the high prevalence rates of anger and aggression in the current sample, if we are to assume that they had experienced traumas that were related to the fact that they were seeking support. The prevalence of anger and aggression might also be explained in part by the military culture in which veterans might have been required to use acts of aggression in their work. Furthermore, most of the participants in this sample were men, and past research has illustrated the existence of a ‘macho’ culture within the armed forces [23], in which the expression of anger and aggression might be a more readily accepted method of displaying emotion.

An association was observed between problems with anger and aggression and higher rates of childhood adversity. Previous large-scale research has shown strong relationships between adverse childhood experiences (ACEs) and an array of difficulties in later life, including for mental health and violent behaviours [24,25]. It has also been suggested that recruits into the military are often from disadvantaged backgrounds [10]. In this study, the links between childhood adversity, anger and aggression were explained by other factors such as severity of PTSD. This perhaps indicates that those with high levels of childhood adversity were more likely to develop PTSD, which in turn was the risk factor for anger and aggression. Indeed, there are some commonalities between anger and PTSD symptoms, such as irritability and hypervigilance which could make some more prone to acting aggressively.

The finding that those who were unemployed due to ill health were more likely to report anger and aggression was notable, particularly because those who were unemployed not due to ill health did not report such difficulties. This could in part be explained if the illnesses in question were related to mental health, given the association here between anger, aggression and mental health. Similarly, those who had the most severe interference with day-to-day functioning might be those who have the biggest mental health difficulties which might explain the association between WSAS scores and anger and aggression.

Participants were more likely to report problems with aggressive behaviour if they did not feel supported by their family. Past research has suggested that social support can help to reduce anger in people with PTSD [26], although the results in the present study relate to aggression, not anger. The fact that perceived support of family but not friends was significant here, could suggest that there is something important about families in the role of aggression in the context of treatment-seeking veterans. Similar findings have been reported elsewhere in a sample of POWs [27], and in an adolescent sample, where anger expression was more likely in those who did not perceive support from family, which was not replicated for support from friends [28]. There is evidence for the notion that social support and family support are important in overcoming PTSD and other mental health difficulties [29,30,31,32], plus there is evidence that social support improves treatment efficacy for PTSD [33]. This finding suggests that those who are not in a relationship or do not feel supported by family are more likely to act on their anger. It may be that close family support is a protective factor stopping some from acting out on their anger, or that those who do not perceive support have been alienated from partners or family members due to their aggressive behaviours.

Results here suggested that women were less likely to report difficulties with anger, although only a small minority of this sample were female. Common narratives exist around the increased likelihood of men to feel anger and act out aggressively, which is supported by some research [34,35], although evidence in some instances is mixed [36].

### 4.1. Limitations

Due to the cross-sectional design of this study, it is not possible to determine causality relating to the associations found. For example, is it the case that veterans display aggression because they do not have support from family, or is it the case that their aggression has caused strain in family relationships? With other variables, such as childhood adversity, we know little from the present study about the mechanisms by which this might relate to anger and aggression. Although, theories from elsewhere in the wider literature might offer suitable explanations, such as the role of childhood adversity on the development of emotional regulation [37]. This study found that some variables were associated with anger but not aggression, or vice versa. It was beyond the scope of the present study to investigate why this occurred, and future research might adopt different designs such as qualitative methods in order to explore this. Furthermore, some evidence suggests an important neurobiological role in PTSD which could help explain the role of some of these variables [38].

The current sample was taken from a population of veterans who were actively seeking treatment from a national veterans mental health charity. CS receives approximately 2500 new referrals per year [12], so the current sample represents a significant number of treatment-seekers but may not be generalisable to all. While the response rate was high, the present study did not conduct a power calculation to determine a sample size required to find significant differences in the given analyses. The relatively large confidence intervals in some of the statistics may point to a lack of power in some instances.

### 4.2. Implications

Findings from the present study suggest that anger and aggression are a significant part of the difficulties faced by the treatment-seeking veteran population. Also, both anger and aggression are strongly related to other comorbid mental health difficulties, such as PTSD. This could be important in identifying mental health difficulties in veterans, if for example a veteran presents with anger, this could be used as a starting point to discuss other difficulties they may be experiencing. Research has shown that there can be a long gap between a veteran completing military service and seeking help for mental health [39], so increasing our knowledge of the main signs and symptoms could help increase the number who are identified and then able to access appropriate support. These findings suggest that anger and aggression should be routinely screened for in mental health assessments of veterans and appropriate treatments offered. Also, it may be pertinent in mental health settings to assess for risk of aggressive behaviours.

## 5. Conclusions

This study showed that veterans who are seeking support with their mental health are likely to be having significant difficulties with anger and aggression, especially if they have other comorbid mental health difficulties. Being unemployed due to ill health, and a lack of perceived family support were also related to higher levels of anger and/or aggression. Being female and over 55 years old were associated with reduced anger and aggression respectively.

Given the high prevalence rates of anger and aggression in treatment-seeking veterans, there is a need to ensure that appropriate forms of assessment and support are available, and the presence of anger or aggression could provide a useful bridge for discussing wider mental health difficulties, given their strong association. While the present study is limited by its cross-sectional design, it provides useful insights into the needs of this population.

## Figures and Tables

**Table 1:** Factors associated with anger and aggression.

Demographic Variable	Anger (DAR-5)		Aggression (WR-4)	
	*n* (%)	OR (95% CI)	*n* (%)	OR (95% CI)
*Sex*				
Male	286 (75.1)	1.00	112 (29.0)	1.00
Female	8 (47.1)	0.34 (0.12–0.92) *	2 (11.8)	0.38 (0.08–1.73)
*Age group*				
<35	37 (75.5)	1.00	21 (42.9)	1.00
35–44	72 (76.6)	1.10 (0.48–2.54)	27 (28.4)	0.54 (0.26–1.14)
45–54	83 (76.9)	1.19 (0.52–2.75)	36 (32.7)	0.68 (0.33–1.42)
55+	102 (69.4)	0.80 (0.36–1.81)	30 (20.1)	0.34 (0.16–0.72) *
*Years to seek help*				
<5 years	153 (72.9)	1.00	58 (27.6)	1.00
>5 years	141 (75.0)	1.25 (0.76–2.07)	56 (29.0)	1.35 (0.84–2.19)
*Childhood adversity*				
Low group	214 (69.5)	1.00	75 (24.0)	1.00
High group	80 (88.9)	3.43 (1.68–7.00) *	39 (43.3)	2.22 (1.34–3.69) *
*Early service leaver*				
No	262 (74.0)	1.00	100 (28.0)	1.00
Yes	32 (72.7)	0.89 (0.42–1.88)	14 (30.4)	1.00 (0.49–2.04)

Note. * *p* ≤ 0.05. OR = Odds Ratio. 95% CI = 95% Confidence Intervals. Odds Ratios adjusted for all other variables in table.

**Table 2:** Associations between factors related to functioning and anger and aggression.

Functioning Variable	Anger (DAR-5)		Aggression (WR-4)	
	*n* (%)	OR (95% CI)	*n* (%)	OR (95% CI)
*Feeling supported by friends*				
Yes	195 (73.3)	1.00	77 (28.6)	1.00
No	76 (81.7)	1.69 (0.90–3.16)	30 (31.9	1.16 (0.69–1.97)
*Feeling supported by family*				
Yes	238 (72.3)	1.00	83 (25.0)	1.00
No	38 (82.6)	1.97 (0.86–4.51)	23 (50.0)	3.10 (1.59–6.01) *
*Relationship Status*				
In relationship	196 (72.9)	1.00	69 (25.3)	1.00
Single	98 (76.0)	1.37 (0.81–2.31)	45 (34.6)	1.62 (1.01–2.62) *
*Employment status*				
Working	83 (67.5)	1.00	27 (21.4)	1.00
Not working	65 (65.7)	0.97 (0.50–1.89)	20 (20.0)	1.31 (0.64–2.68)
Ill not working	146 (83.0)	2.80 (1.58–4.96) *	67 (37.9)	2.62 (1.51–4.55) *
*Functional impairment* (*WSAS*)				
Mild/moderate	80 (59.7)	1.00	28 (20.7)	1.00
Severe (21+)	214 (81.1)	2.89 (1.77–4.74) *	86 (32.1)	1.01 (1.00–2.79) *

Note. * *p* ≤ 0.05. OR = Odds Ratio. 95% CI = 95% Confidence Intervals. Odds Ratios adjusted for age, sex and childhood adversity.

**Table 3:** Health factors associated with anger and aggression.

Health Variable	Anger (DAR-5)		Aggression (WR-4)	
	*n* (%)	OR (95% CI)	*n* (%)	OR (95% CI)
*PTSD* (*PCL-5*)				
Not a case	23 (31.9)	1.00	4 (5.6)	1.00
Case (38+)	271 (83.1)	10.7 (5.79–19.6) *	110 (33.2)	8.71 (2.99–25.4) *
*CMD* (*GHQ-12*)				
Not a case	60 (55.1)	1.00	11 (10.0)	1.00
Case (4+)	234 (81.0)	4.14 (2.47–6.94) *	103 (35.5)	6.00 (2.97–12.1) *
*Alcohol* (*AUDIT*)				
Not a case	225 (71.2)	1.00	79 (24.8)	1.00
Case (16+)	69 (84.2)	2.08 (1.08–4.01) *	35 (41.7)	2.05 (1.21–3.47) *
*Brain Injury*				
Negative	148 (71.5)	1.00	58 (27.5)	1.00
Positive	146 (76.4)	(1.24 (0.78–1.98)	56 (29.2)	1.04 (0.66–1.65)

* *p* ≤ 0.05.

**Table 4:** Exploring associations with anger and aggression adjusting for all other significant variables previously identified.

Variable	Anger (DAR-5)	Aggression (WR-4)
	OR (95% CI)	OR (95% CI)
*Sex*		
Male	1.00	1.00
Female	0.20 (0.06–0.65) *	0.32 (0.07–1.57)
*Age group*		
<35	1.00	1.00
35–44	1.38 (0.54–3.54)	0.59 (0.26–1.32)
45–54	1.14 (0.46–2.86)	0.59 (0.27–1.28)
55+	0.98 (0.39–2.46)	0.31 (0.14–0.70) *
*Childhood adversity*		
Low group	1.00	1.00
High group	1.35 (0.72–2.53)	1.37 (0.81–2.33)
*Feeling supported by family*		
Yes	1.00	1.00
No	1.36 (0.52–3.53)	2.54 (1.20–5.36) *
*Relationship status*		
In relationship	1.00	
Single	0.88 (0.47–1.66)	0.85 (0.47–1.53)
*Employment status*		
Working	1.00	1.00
Not working	0.88 (0.40–1.94)	1.34 (0.60–3.03)
Ill not working	1.31 (0.66–2.60)	1.94 (1.05–3.59) *
*Functional impairment* (*WSAS*)		
Mild/moderate	1.00	1.00
Severe	1.64 (0.91–2.95)	1.11 (0.61–2.03)
*PTSD* (*PCL-5*)		
Not a case	1.00	1.00
Case (38+)	6.06 (3.12–11.77) *	3.45 (1.14–10.43) *
*CMD* (*GHQ-12*)		
Not a case	1.00	1.00
Case (4+)	1.87 (1.03–3.38) *	3.35 (1.63–6.90) *
*Alcohol* (*AUDIT*)		
Not a case	1.00	1.00
Case (16+)	1.51 (0.73–3.13)	1.83 (1.02–3.28) *

Note. * *p* ≤ 0.05. CMD = Common Mental Health Disorders. OR = Odds Ratio. 95% CI = 95% Confidence Intervals. Odds Ratios adjusted for age, sex, childhood adversity, family support, relationship status, employment status, functional impairment (WSAS), post-traumatic stress disorder (PTSD; PCL-5), CMD (GHQ-12), Alcohol (AUDIT).
